# The intricate interactions between the lungs and gut in patients: unraveling the crosstalk mechanism

**DOI:** 10.3389/fmed.2025.1624907

**Published:** 2025-07-30

**Authors:** Jiale Li, Yiting Chen, Bangchuan Hu

**Affiliations:** ^1^The Second Clinical Medical College, Zhejiang Chinese Medical University, Hangzhou, China; ^2^Emergency and Critical Care Center, ICU, Zhejiang Provincial People’s Hospital (Affiliated People’s Hospital, Hangzhou Medical College), Hangzhou, Zhejiang, China

**Keywords:** lung-gut crosstalk, ventilator-induced lung injury, inflammatory bowel disease, immunity, microbiome, adipokines

## Abstract

There is a growing body of evidence indicating that the stimulation of one organ can significantly influence the functioning of another. For instance, intestinal complications are frequently observed during respiratory diseases, and conversely, pulmonary complications can arise during intestinal diseases—a phenomenon referred to as lung-gut crosstalk. Patients suffering from mechanical ventilator-induced lung injury, chronic obstructive pulmonary disease (COPD), acute respiratory distress syndrome (ARDS), and other pulmonary conditions have been shown to experience gastrointestinal dysfunction and related disorders. Similarly, individuals with inflammatory bowel disease (IBD) have also been found to develop pulmonary complications. However, these studies are not enough to fully explain the mechanism of lung-intestinal crosstalk, and more potential mechanisms need to be explored and further elucidated. In this paper, we summarize recent research advancements regarding lung-intestinal interactions in the context of pulmonary and intestinal diseases, analyzing the potential mechanisms of lung-intestinal crosstalk from the perspectives of respiratory mechanics, inflammation, and microbiota. Additionally, we review evidence suggesting that adipokines may play a role in lung-gut interactions, and we propose new avenues for investigating the mechanisms underlying these interactions.

## 1 Introduction

As early as 1968, Warwick introduced the concept of lung-gut interaction, providing significant evidence for this phenomenon (1). With advancements in technology and improvements in clinical practices, it has been observed that patients with mechanical ventilation, lung injury, and lung diseases such as chronic obstructive pulmonary disease (COPD) and acute respiratory distress syndrome (ARDS) exhibit a higher incidence of gastrointestinal disorders. Conversely, respiratory diseases have also been identified in patients suffering from inflammatory bowel disease (2). These findings indicate a profound physiological and pathological relationship between the respiratory system and the intestine. Despite the increasing body of evidence elucidating lung-gut interaction, the underlying mechanisms remain poorly understood. Several population-based and observational studies have revealed an increased prevalence of respiratory diseases among patients with IBD, including ulcerative colitis (UC) and Crohn’s disease (CD), even in non-hospitalized settings. For instance, a nationwide cohort study reported that individuals with IBD had higher risks of developing asthma, bronchiectasis, and COPD, independent of smoking status and other confounders (3, 4). Conversely, individuals with chronic respiratory diseases such as asthma and COPD also exhibit higher incidences of IBD compared to the general population (5, 6). These findings indicate that the lung-gut axis interaction is not limited to critically ill or ventilated patients but may exist as a broader phenomenon affecting immunologically and microbially susceptible populations. The lung–gut axis has been increasingly recognized as a crucial mediator of pulmonary immune responses. Recent studies have elucidated the bidirectional interactions between gut microbiota and pulmonary inflammation in various diseases including lung cancer, asthma, and infections (7, 8). Such evidence underscores the importance of further investigating the mechanisms linking these two systems. In this paper, the authors analyze the mechanisms linking pulmonary and intestinal diseases to enhance our understanding of lung-intestinal interaction. Additionally, we explore the role of adipokines in this interaction, offering new insights into its effects.

## 2 Evidence for the existence of lung-gut crosstalk

To build the foundation for understanding the lung-gut axis, we first summarize clinical and experimental evidence demonstrating bidirectional interactions between pulmonary and intestinal systems. The lungs and intestines originate from the anterior region of the intestinal endoderm. Although they are anatomically separate, stimulation of one organ affects the other compartments, suggesting important communication between the gut and lungs.

### 2.1 From the lung to gut

The lung can influence intestinal function through multiple pathways, particularly under conditions of acute or chronic respiratory disease. Various lung diseases, including Ventilator-Induced Lung Injury (VILI), COPD, and respiratory tract infections caused by viruses or bacteria, can lead to gastrointestinal disease or dysfunction (9–11). VILI refers to the acute lung damage caused or worsened by mechanical ventilation. This type of injury can occur during both invasive and non-invasive ventilation and has been shown to play a significant role in the morbidity and mortality of critically ill patients (12). The primary mechanisms responsible for ventilator-induced lung injury are alveolar overdistention (volutrauma), barotrauma, atelectotrauma, and inflammation (biotrauma). Additional contributing factors include adverse heart-lung interactions, deflation-related injuries, and effort-induced injuries (13). While mechanical ventilation causes lung damage, it is also accompanied by changes in intestinal diseases. In a previous retrospective analysis, a study included a total of 242 patients, with 113 (46.7%) experiencing gastrointestinal bleeding. Among these patients, 86 had coffee ground material or positive occult blood in nasogastric aspirates, 12 had positive occult blood in stools, 5 had hematemesis, 2 had hematochezia, and 2 had both hematemesis and hematochezia. Clinically significant bleeding was diagnosed in eight patients (3.3%) with gastrointestinal bleeding. The majority of patients (67.3%) developed gastrointestinal bleeding within the first 48 h of mechanical ventilation, and 80% experienced it within the first 2 weeks (11).

However, the causes of COPD are often associated with long-term smoking and inhaling air pollutants. Its main features include persistent airflow obstruction and respiratory symptoms. Smoking is considered an independent risk factor for COPD and various gastrointestinal diseases such as Crohn’s disease (CD) (14). Multiple studies have shown that smoking may increase the risk of Crohn’s disease by three times (15). In a recent study, smoking was found to be associated with several functional gastrointestinal disorders and functional symptoms such as functional bloating, functional abdominal pain, functional diarrhea, and functional constipation (16). A population-based cohort study by Ekbom et al. (17) revealed that COPD patients had a 2.72% higher risk of developing CD and a 1.83% higher risk of developing ulcerative colitis (UC) compared to the control group (17). Additionally, a previous questionnaire-based prospective study showed that the highest proportion of patients with maximal gastrointestinal (GI) symptoms was found in the “GOLD-4” group of patients with COPD and the “uncontrolled” group of patients with asthma. From this, we can see that not only does lung-gut crosstalk exist, but the severity of symptoms in one system (bowel) is highly consistent with the severity of the other system (lung) (15).

In intensive care units, sepsis is the main indirect cause of acute lung injury and acute respiratory distress syndrome (18). Although the pathogenesis of sepsis, acute lung injury (ALI), and ARDS is complex and multifactorial, there are still some phenomena that support the interaction between lung and gut. In clinical cases, the most common source of sepsis is the lung (19). But during antibiotic treatment, the intestinal flora is also affected, resulting in microbial imbalance. Rosa discovered that Vancomycin treatment for acute Pseudomonas aeruginosa pneumonia in mice can lead to intestinal dysbacteriosis, causing an increase in Proteus and a decrease in Bacteroides, along with inflammatory changes in the intestinal tract (20). Following fecal microbiota transplantation, the susceptible and tissue injury phenotypes were reversed in mice. Additionally, pulmonary allergic responses impact the composition of the intestinal microbiota (21). In experimental influenza infection, it has been observed that IFN-γ produced by lung-derived CCR9 + CD4 + T cells alter the gut microbiota composition and induce intestinal immune injury (22). Furthermore, pulmonary IFN-Is production promotes the depletion of obligate anaerobic bacteria and the proliferation of proteobacteria in the gut, leading to significant intestinal dysregulation (23). Therefore, lung inflammation directly influences the structure of the intestinal bacterial community, exacerbating lung inflammation.

### 2.2 From the gut to lung

Conversely, gastrointestinal inflammation, especially in IBD, has been shown to contribute to pulmonary manifestations, highlighting the bidirectional nature of the lung-gut axis. When it comes to the impact of intestinal diseases on the lungs, inflammatory bowel disease is a very typical condition that causes lung dysfunction. IBD is a term used to describe chronic recurring inflammation in the gastrointestinal tract, with the major types being Crohn’s disease (CD) and ulcerative colitis (UC). CD is identified by transmural non-continuous and non-caseating granulomatous inflammation that can occur anywhere in the gastrointestinal tract but is most frequently found in the terminal ileum (24, 25). On the other hand, UC is characterized by continuous inflammation starting in the rectum and spreading upwards. Unlike CD, UC inflammation only affects the mucosa and submucosa, specifically in the colon (26, 27). Studies suggest that 40–60% of patients with IBD exhibit subclinical lung complications, which can be detected through changes in pulmonary function tests and high-resolution tomographic imaging (HRCT) (28). With the increasing use of bronchoscopy and histological examinations, a growing number of patients with IBD are being identified as having potential pulmonary dysfunction even in the absence of clear symptoms (29, 30). Various aspects of pulmonary involvement in IBD have been documented in the literature. For instance, Camus observed that out of 33 patients with ulcerative colitis (UC) or Crohn’s disease (CD), 60% exhibited bronchiectasis, inflammatory/obliterative small airway lesions, or upper airway narrowing (31). In a review conducted by Desai, it was found that bronchiectasis was present in 45% of IBD cases with large airway involvement (32). Furthermore, a population-based cohort study revealed that individuals with IBD, specifically UC and CD, had a higher prevalence of bronchiectasis (46%), pulmonary vasculitis, and interstitial pneumonia (52%), lung nodules (35%), pulmonary fibrosis (16%), and asthma (5.5%) compared to those without IBD (33). A recent survey based on a questionnaire of 13,499 participants showed that IBD patients, particularly those with ulcerative colitis and women, had a higher prevalence of asthma and respiratory symptoms (34). Therefore, from the perspective of lung disease caused by IBD, the phenomenon of gut-lung interaction has obvious manifestations.

### 2.3 Hypoxia and intestinal injury in pulmonary disease

Hypoxia and hypoxemia associated with pulmonary diseases represent a crucial pathway mediating lung-gut interaction. Systemic hypoxia can disrupt intestinal homeostasis by impairing epithelial oxygenation, leading to ATP depletion, increased intestinal epithelial apoptosis, and compromised barrier function. Hypoxia-inducible factors (HIFs), particularly HIF-1α, play dual roles in maintaining mucosal integrity and mediating inflammation, depending on the duration and severity of hypoxic stress (35, 36). Moreover, oxygen deprivation alters the gut microbial ecology, favoring pro-inflammatory species and increasing the risk of bacterial translocation (37, 38). These mechanisms explain the occurrence of gastrointestinal dysfunction in patients with severe respiratory diseases, even in the absence of mechanical ventilation.

## 3 Mechanisms of pulmonary disease in lung-gut crosstalk: patients with mechanical ventilation

### 3.1 Impact of mechanical ventilation from a mechanistic point of view

**FIGURE 1:**
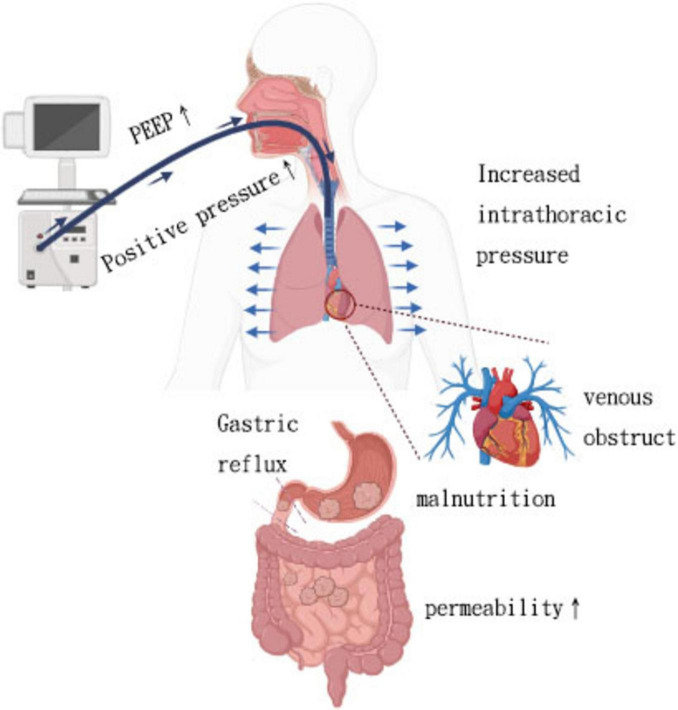
Interaction between mechanical ventilation and its effects on both lung and gut.

Beyond epidemiologic observations, mechanistic studies help elucidate how mechanical ventilation physiologically influences gut function. Mechanical ventilation involves the use of a ventilator, a medical device frequently utilized in intensive care units to support respiratory function, optimize the exchange of gases, and prevent hypoxia and hypercapnia. Several experimental studies in humans and animals have explored the MV-intestinal interactions from a mechanistic perspective. Positive pressure ventilation increases intrathoracic pressure, leading to decreased venous return, cardiac preload, and cardiac output, ultimately resulting in hypotension. Animal experiments have shown that splanchnic blood flow decreases in tandem with reductions in cardiac output induced by mechanical ventilation, even when mean arterial pressure is maintained (39). Additionally, gastric mucosal ischemia due to hypoperfusion is a common cause of gastrointestinal bleeding in mechanically ventilated patients. A retrospective study of 283 ICU patients ventilated for over 48 h found that peak inspiratory pressure ≥ 30 cm H_2_O was an independent risk factor for gastrointestinal bleeding in this population (11). However, PEEP can autonomously impact regional perfusion, with a particular focus on hindering splanchnic perfusion. This hindrance in splanchnic perfusion may lead to compromised gut barrier function, resulting in intestinal hyperpermeability, bacterial translocation, and ultimately facilitating the onset of multiple organ failure (MOF) (Figure 1) (40). Experimental studies have demonstrated a dose-dependent relationship between PEEP and splanchnic blood flow. Splanchnic blood flow reduction is typically limited at PEEP levels below 10 cm H_2_O but becomes more pronounced within the range of 15–20 cm H_2_O (41).

Positive pressure ventilation with high PEEP, while improving arterial oxygenation, can lead to blocked venous return, causing gastrointestinal tract congestion, edema, and feeding intolerance. In a recent retrospective study of 97 adult patients, Ryuichiro et al. found that high central venous pressure (CVP) during the first week after left ventricular assist device implantation was an independent risk factor associated with decreased enteral nutrition (EN) calorie intake (42).

The transmission of thoracic-abdominal pressure results in an increase in intra-abdominal pressure due to mechanical ventilation. Previous studies have also indicated that ventilatory-related pressure parameters may exacerbate gastrointestinal dysfunction by impacting intra-abdominal pressure (43, 44). Controlled mechanical ventilation results in increased internal abdominal pressure, causing the diaphragm to ascend. Prolonged mechanical ventilation can result in phrenic atrophy. Animal studies have demonstrated that even short durations of controlled mechanical ventilation, such as 18 h, can lead to diaphragm atrophy and weakness (45). This reduced ability of the diaphragm to contract may result in reflux of stomach contents, increasing the risk of gastroesophageal reflux for patients. Training the diaphragm of COVID-19 patients has been shown in previous studies to significantly reduce the risk of gastroesophageal reflux (46). Meanwhile, the possibility of gastrointestinal flora entering the lungs decreases.

In addition, commonly used drugs that support mechanical ventilation, such as opioids and sedatives (especially benzodiazepines), may reduce gastrointestinal motility and venous reflux through mechanisms such as venodilation or reduced response to vasopressors (47).

### 3.2 Impact of mechanical ventilation from an immunity point of view

The effects of mechanical ventilation extend beyond hemodynamics and include profound immunologic consequences, both locally in the lung and systemically, with implications for gut barrier integrity. Mechanical ventilation not only supplies the patient with oxygen, but it can also lead to lung injury (LI) due to increased inflation transpulmonary pressures (barotrauma), excessive alveolar distension (volutrauma), and the repetitive opening and closing of alveoli (atelectrauma). In addition to causing direct structural changes, these mechanical forces can also initiate an intricate inflammatory process, leading to inflammation at both local and systemic levels (biotrauma) (13). This inflammation has the potential to spread to other organs and systems, worsening multiple organ dysfunction and ultimately increasing mortality. Mechanical ventilation can potentially induce an inflammatory response and lead to lung damage, despite its effectiveness in maintaining gas exchange. These inflammatory processes may spread through the bloodstream, affecting organs outside the lungs and contributing to multiple organ failure (MOF).

It is believed that by increasing the permeability between alveoli and blood vessels, mechanical ventilation plays a role in expanding the inflammatory response beyond the lungs, ultimately causing damage to distant organs and impacting mortality rates (48). The inflammatory response induced by mechanical ventilation (MV), also known as biotrauma, is attributed to two distinct pathophysiologic mechanisms.

The first mechanism involves direct cellular trauma that disrupts cell walls, leading to the release of cytokines both locally at the alveolar level and systemically (49). The second mechanism, termed “mechanotransduction”, describes how cells detect mechanical forces through mechanosensation and convert them into cellular signaling events through mechanotransduction. *In vitro* studies have shown that a majority of pulmonary cells can produce cytokines in response to cyclic stretch (50). Mechanical injury triggers danger-associated molecular patterns (DAMPs) release, recruiting immune cells and inducing pro-inflammatory cytokine production. This, along with signaling cascade activation in alveolar epithelial and vascular endothelial cells from overstretching, as well as dysregulation of the neuroinflammatory reflex, results in a robust systemic inflammatory response. Prolonged exposure to harmful mechanical forces, especially high tidal volume MV, exacerbates this inflammatory process, leading to heightened alveolar-capillary barrier permeability and increased susceptibility to further lung damage (51).

Previous experimental studies have demonstrated that mechanical ventilation can induce the release of several pro-inflammatory mediators such as IL-1β, IL-6, IL-8, TNF-α, C-X-C motif ligand 1 (CXCL1), and CXCL10 (52). Meanwhile, elevated levels of TNF-α, IL-1β, and IL-6 as part of the inflammatory/sepsis cascade have been found to affect the permeability of tight junctions (TJ) in the intestine (53).

A team demonstrated that mechanical ventilation (MV) increases plasma TNF levels and gut permeability. They also found that administering a TNF-neutralizing antibody intravenously reversed gut hyperpermeability (54). Previous studies have highlighted the negative impact of TNF-α on the integrity of the intestinal epithelial barrier, either alone or in combination with other proinflammatory cytokines in various experimental models and cell culture settings (55–58). The mechanisms through which TNF-α disrupts the intestinal epithelial barrier involve multiple processes, such as inducing apoptosis in intestinal epithelial cells, changing the lipid composition of cell membranes, activating MLCK via calcium-calmodulin, promoting myosin light chain (MLC) phosphorylation by increasing MLCK protein expression, and reducing TJ protein expression. Particularly, the MLCK-mediated MLC phosphorylation pathway is crucial in the development of TNF-α-induced dysfunction in the intestinal epithelial barrier (59). In addition, IL-6 is a multifunctional inflammatory cytokine shown to participate in local and systemic inflammatory responses in the intestine, with pleiotropic effects. Whether IL-6 plays a protective role or disrupts the intestinal barrier is controversial (53). Recently, Ding used an experimental mice model of VILI to show increased levels of TNF-α, IL-1β, and IL-6 in both serum and gut tissues, measured by enzyme-linked immunosorbent assay (ELISA) (60). Additionally, Imai found that harmful ventilatory strategies, like high TV and zero PEEP, significantly increased epithelial cell apoptosis in the kidneys and small intestine, which correlated with organ dysfunction (61).

### 3.3 Impact of mechanical ventilation from a microbiome point of view

Another critical dimension of lung-gut interaction lies in the disruption of the respiratory and gastrointestinal microbiomes under mechanical ventilation. Utilizing culture-independent molecular techniques, researchers have identified the presence of non-pathogenic anaerobes such as Prevotella, Veillonella, and Fusobacterium in the normal alveoli of humans, which likely originate from the oropharyngeal flora (62–64). These lower levels of bacteria are believed to contribute to the maintenance of the ecological balance within the lungs (65).

In certain critical situations, patients in the ICU may require various interventions, including mechanical ventilation, antibiotic therapy, continuous blood purification, and immunosuppressive regimens (66). These interventions have the potential to impact the microbial composition and diversity of these patients (67–69). Changes in the lung microbiome due to critical illness are closely linked to both bacterial translocation, systemic, and local inflammation (62, 67, 70–72). The commensal microbial populations in the lungs may be displaced by potential pathogens from other ecosystems, such as the gastrointestinal tract and the skin (62, 73). In the study of BALF from ARDS patients, Kyo found an increase in lung bacterial burden as shown by elevated 16S rRNA gene copy numbers. They also noted a decrease in alpha diversity among ARDS patients, affecting both copy numbers and the relative abundance of betaproteobacteria (74). Additionally, patients with LI show higher levels of gut-associated bacteria in their lung microbiome, including species from the Enterobacteriaceae family, which have been linked to the development of ARDS (71).

Observational studies have suggested that gut microbes were not present in the upper airways of intubated individuals with ARDS or in septic mice, indicating bacterial translocation rather than aspiration. Panzer emphasizes that patients who undergo mechanical ventilation (MV) may experience early lung dysbiosis, which is accompanied by a significant rise in inflammatory mediators (IL-6, IL-8). This situation predisposes patients to the development of ARDS later (70). The movement of bacteria may occur through gut-draining lymphatics, the portal system, or systemic circulation (75, 76).

Limited data exists on how lung dysbiosis may impact the gut microbiome in patients with LI/ARDS. The compromised permeability of the alveoli-capillary membrane in LI/ARDS caused by epithelial or endothelial damage may contribute to gut-lung bacterial translocation (77). Critical illness can also alter environmental conditions that support gut bacterial growth, affecting the reproductive rates of microbial community members (67). In pulmonary infection diseases, microorganisms originating from the lungs trigger a pro-inflammatory response in intestinal epithelial cells, causing an increase in TJ permeability and damage to intestinal mucosal integrity. This ultimately leads to gastrointestinal injury and the development of MODS (78, 79).

There is increasing evidence suggesting that alterations in the lung microbiome can impact gut integrity, and the translocation of gut microbiota may worsen lung infections. However, there are significant gaps in knowledge regarding how lung dysbiosis during mechanical ventilation specifically influences the gut microbiome. Further research is crucial to uncover these mechanisms and their importance in clinical settings.

## 4 Potential mechanisms linking inflammatory bowel disease to pulmonary dysregulation

### 4.1 Dysbiosis of intestinal microbiota and inflammation in IBD

**FIGURE 2:**
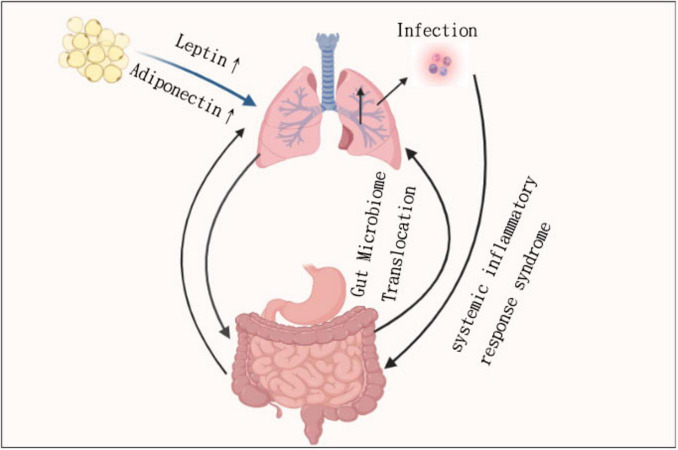
The core pathways between lung inflammation and gut permeability, including dysbiosis and IBD risk.

Typically, dysbiosis in patients with IBD is associated with changes in increased pro-inflammatory bacteria as well as reduced protective bacteria. In patients with Crohn’s disease (CD), intestinal dysbiosis, characterized by reduced diversity of Firmicutes species, is thought to occur before disease onset (80–82). A specific reduction in the relative abundance of the species Dialister invisus and Faecalibacterium prausnitzii from the Firmicutes phylum, as well as Bifidobacterium adolescentis from the Actinobacteria phylum, is observed in patients with Crohn’s disease (CD) compared to healthy controls. In contrast, there is an increase in the mucolytic species Ruminococcus gnavus and Ruminococcus torques, also belonging to the Firmicutes phylum, among CD patients (80, 83). Common experimental mouse models support the importance of the gut microbiota in the development of IBD, such as protein 2 (NOD2) deficient in the nucleotide-binding oligomerization domain, which typically has ileitis under standard feeding conditions. However, disease penetrance is reduced in certain pathogen-free environments (84–86) (Figure 2).

Adherent invasive Escherichia coli (AIEC) is an organism commonly associated with CD. This bacterium can exploit weaknesses in host defenses, such as impaired bacterial recognition, thereby reducing intracellular killing capacity and allowing AIEC dissemination (87). AIEC can survive and multiply in macrophages without causing cell death. This process also promotes increased secretion of the proinflammatory tumor necrosis factor alpha (TNF-α), which plays a role in Crohn’s disease (88).

A study in an inducible model of ileitis found that severe ileitis was associated with a shift in microbial communities. This shift transitioned from populations predominantly composed of species within the Firmicutes phylum to those largely represented by species within the Proteobacteria phylum. The translocation of AIEC is associated with dysbiosis that resembles that observed in patients with CD. Toxoplasma gondii induces a significant degree of dysbiosis with AIEC invasion being pronounced in mice deficient in the ileitis susceptibility gene NOD2, while it is notably reduced in mice lacking the pro-inflammatory CC chemokine receptor 2 (CCR2). This model illustrates the mechanisms of resistance to enteritis (89). Furthermore, in alternative models of ileitis susceptibility, dysbiosis has been shown to precede the development of ileitis (90).

Similar to CD, UC is characterized by a decrease in the number of Firmicutes species and an increase in the number of Proteobacteria species. UC is also related to the interaction of genetic susceptibility, inflammation, and microbial disturbances (91).

### 4.2 Intestinal dysbiosis influences lung health

Although direct experimental evidence remains limited, emerging research suggests that alterations in the intestinal microbiota may impact pulmonary immunity and inflammation. These proposed gut-lung connections are primarily derived from animal studies and correlational data in human cohorts, highlighting the need for further mechanistic research. Studies demonstrate that when antibiotic treatment eradicates gut bacteria, mice exhibit increased susceptibility to pneumonia and respiratory infections. In the absence of gut microbiota, alveolar macrophages experience transcriptomic changes that result in diminished phagocytic activity and reduced bacterial clearance (92, 93). Through changes in circulating inflammatory cytokines and changes in the airway of the intestinal microbiota, it has been demonstrated that intestinal microbiota dysbiosis affects the composition of the respiratory microbiota. This has only been demonstrated in models of severe sepsis, but not yet in COPD.

One study showed that segmented filamentous bacteria (SFB) in the gut induce Th17 responses and IL-22 production in the lungs and prevent pneumococcal respiratory infections by reducing bacterial numbers and lung inflammation (94). In a mouse model of sepsis, more abundant gut bacteria were observed in the lungs, and the communities found in the gut and lungs were more similar compared to sham mice (70). A meta-analysis of 16 studies found that Helicobacter pylori infection is associated with an increased risk of COPD (95). Helicobacter pylori is a bacterium that resides on the lining of the human stomach. During acute exacerbations of COPD, the gut microbiota exhibits a significant reduction in bacterial classes defined by operational taxonomic units (OTUs) (9). Although the gut microbiota has not been extensively studied in COPD, smoking—an independent risk factor for the condition—exhibits a strong association with COPD. Additionally, smoking is linked to dysbiosis in the fecal microbiota of patients with Crohn’s disease (CD), characterized by an increased relative abundance of Bacteroides and Prevotella (96).

### 4.3 Immunological crosstalk in the lung–gut axis

Immunological signaling is central to the bidirectional communication between the lungs and the gut. Mucosal barriers in both organs are densely populated by innate and adaptive immune cells, including macrophages, dendritic cells (DCs), innate lymphoid cells (ILCs), and T lymphocytes, which play crucial roles in maintaining homeostasis and orchestrating inflammatory responses (97, 98).

During respiratory infections or mechanical injury (e.g., mechanical ventilation), activated alveolar macrophages release cytokines such as IL-6, TNF-α, and IL-1β, which may enter systemic circulation and influence intestinal immune tone (99, 100). Similarly, in IBD, elevated levels of IL-17, IL-23, and other pro-inflammatory cytokines contribute to lung injury and immune cell infiltration in the airway.

Furthermore, the gut microbiota indirectly shapes pulmonary immunity by modulating the differentiation and migration of immune cells. Short-chain fatty acids (SCFAs) derived from gut commensals promote regulatory T cell (Treg) development and can suppress excessive inflammation in the lung (101, 102). Disruption of the microbiome, therefore, may impair mucosal immune tolerance and promote pathological inflammation in both organs.

Of particular interest is the role of the gut–lung axis in systemic immune priming. Studies have demonstrated that antigen-presenting cells in the gut can prime T cells that later home to the lungs during allergic asthma or bacterial pneumonia, highlighting a shared mucosal immunological network (103).

Collectively, these findings support that the lung–gut axis functions as an integrated mucosal immune circuit, capable of driving both protective and pathogenic outcomes depending on the immune context and disease stage.

### 4.4 Alterations in the lung microbiome in pulmonary diseases

While much attention has focused on gut microbiota, the composition of the lung microbiome also plays a critical role in the lung–gut axis. Unlike the sterile lung paradigm once believed, it is now established that healthy lungs harbor low-biomass but functionally significant microbial communities.

In disease states, the pulmonary microbiota undergoes notable alterations. In COPD and asthma, there is a shift toward increased Proteobacteria (e.g., Haemophilus influenzae) and decreased diversity (104). In ARDS, lung microbiota shows enrichment in gut-associated taxa, suggesting possible translocation and systemic dissemination during severe lung injury (105). Although gut-derived bacteria have been detected in the lungs of ARDS patients, whether these microbes represent active colonization or transient presence remains unclear. Moreover, direct evidence from metagenomic or metabolomic studies evaluating their functional impact on pulmonary immunity is currently limited, underscoring the need for further multi-omics investigations in this area.

These microbial changes can modulate local and systemic immunity. For instance, enrichment of pathogenic microbes in the lung can promote pro-inflammatory cytokine production, which in turn affects gut permeability and immune tone (106). Conversely, dysbiosis of lung microbiota may reduce regulatory immune signals, leading to enhanced susceptibility to gut inflammation.

Moreover, interactions between pulmonary microbiota and gut microbiota may be mediated through shared metabolites (e.g., SCFAs, bile acids) or systemic immune circuits involving dendritic cells and regulatory T cells (107).

Understanding how lung microbiota shifts in response to infections and chronic inflammation is essential to fully elucidate the bidirectional crosstalk along the lung–gut axis.

## 5 Emerging roles of adipokines in lung–gut crosstalk: hypotheses and early evidence

In addition to mechanical and inflammatory factors, adipose-derived signaling molecules—adipokines—have emerged as potential mediators of lung-gut crosstalk. Adipose tissue is now increasingly recognized as a hormonally active organ capable of influencing both local and systemic inflammation. While the role of adipokines in systemic inflammatory diseases is relatively well-established, their specific contribution to lung–gut crosstalk remains largely hypothetical. Here, we summarize selected findings from human and animal studies that may offer insight into potential mechanisms, while acknowledging that direct evidence remains limited. The endocrine function of adipose tissue depends on its ability to secrete hormones, factors, and protein signals (termed “adipokines”) that are responsible for metabolic and pro-inflammatory effects. In obese patients, the elevated release of pro-inflammatory adipokines has a detrimental impact on lung health. The influence of lung-related inflammatory factors on intestinal function has been previously elucidated, making it crucial to explore the role of adipokines in the lung-intestinal interaction. This discussion will enhance our understanding of the mechanisms underlying the lung-intestinal interplay (108, 109).

Since the discovery of adipokines, adipose tissue has been identified as a central node in inter-organ crosstalk networks that mediate regulation of the respiratory system in multiple organs and tissue types (110). Adipokines are the mediators between adipose tissue and target organs and is a key factor in metabolic disorders caused by obesity (111). Dysfunctional adipose tissue releases inflammatory adipokines in morbid obesity, leading to increased risks of pulmonary dysfunction, resulting in low-grade systemic inflammation (112).

### 5.1 Leptin and adiponectin: linking adipose dysfunction to pulmonary immunity

Leptin and adiponectin, two of the most abundant adipokines, exert profound regulatory effects on pulmonary immune responses and inflammation. Acting through both systemic and local mechanisms—including modulation of surfactant production, macrophage activation, and Treg/Th17 balance—these adipokines bridge the metabolic status of adipose tissue with the development and progression of various respiratory diseases. Understanding their opposing roles is essential for elucidating how adipose dysfunction contributes to lung pathology, especially in obesity-related conditions such as asthma, COPD, and COVID-19 (109, 113–116).

Leptin, a hormone secreted by adipose cells, has its receptors expressed both centrally in the hypothalamus and peripherally in various tissues (117, 118). Additionally, leptin can be produced in the lungs by adipose-like lipofibroblasts, where it plays a crucial role in the regulation of pulmonary surfactant production (119). It has been demonstrated that there is a significant association between genetic variants of the leptin receptor and the decline in lung function among patients with COPD (116). Furthermore, the relationship between leptin and the pathogenesis of lung disease is influenced by excess adipose tissue. Research indicates that serum leptin levels are strongly correlated with both body fat and lung function, and that elevated leptin levels contribute to obesity hypoventilation syndrome (OHS) (120).

Adiponectin is a hormone secreted by adipose tissue that plays a crucial role in the regulation of whole-body energy homeostasis. In contrast to leptin, adiponectin levels are observed to be reduced in individuals with impaired lung function and obesity (121–123).

Both leptin and adiponectin have a direct regulatory effect on adipose tissue function (117, 124) and new biomarker indicators of dysfunctional adipose tissue have been proposed to use the ratio of adiponectin to leptin serum levels (125). There is evidence that dysfunction of adipose tissue caused by obesity and systemic inflammation can cause airway inflammation and lead to lung disease (126). Multiple studies have shown an inverse correlation with the adiponectin/leptin serum ratio in patients with pulmonary dysfunction (127). These findings suggest that altering the adipokine secretion spectrum produced by adipose dysfunction in obese subjects may contribute to the decline of lung function in COVID-19 patients. Similar associations were observed in obese asthmatic patients compared with obese subjects without asthma, in which, in addition to increases in serum markers of inflammation and alterations in alveolar macrophage function, adiponectin/leptin mRNA expression ratio is also observed to be lower in adipose tissue. Bariatric surgery in obese patients with asthma has been shown to reverse inflammation and restore macrophage function, indicating a connection between excess adipose tissue, the pathogenesis of lung disease, and lung immune function (126).

Previous studies have shown that leptin and adiponectin have a direct regulatory effect on lung inflammation and immune function (128–130). Indeed, adiponectin-related adipoR1 has been observed to be expressed in immune regulatory T cells (T cells) in the lungs, regulating immune responses during lung inflammation (131). Regulatory T cells (Tregs) play a crucial role in suppressing immune responses, and their development is closely linked to helper T17 (Th17) cells, which can enhance immune responses and inflammation. Therefore, the balance between these two cell populations is essential for the regulation of the immune system. *In vivo* studies in mice have demonstrated that the activation of adipoR1 in lung Tregs promotes their proliferation and activity, thereby effectively suppressing immune responses and inflammation (128). Leptin has been shown in animal and *in vitro* models to exert opposing effects on regulatory T cells (Tregs) and Th17 cells, potentially impacting inflammatory balance in both pulmonary and intestinal tissues. However, direct confirmation of these effects in the context of lung–gut interaction is still lacking (129). Furthermore, the administration of leptin to Th2 immune cells in the lungs of mice enhanced the production of pro-inflammatory cytokines including IL-4, IL-5, and IL-13, while concurrently reducing allergen-induced inflammation in leptin-deficient mice (130, 131). These findings indicate that obesity-related dysfunction of adipose tissue may modify the adipokine profile, specifically the adiponectin/leptin ratio. This alteration could result in an imbalance in the Treg/Th17 ratio, an enhancement of Th2 responses, and an elevation in proinflammatory cytokine secretion, all of which may contribute to an increased risk of respiratory diseases (132).

### 5.2 Immunological functions of leptin and adiponectin in intestinal inflammation

Characteristic alterations in mesenteric fat associated with Crohn’s disease, a subtype of IBD, indicate that mesenteric fat may have a significant immunomodulatory role in the pathogenesis of this condition. In Crohn’s disease, mesenteric fat proliferates and encircles inflamed regions of the small intestine, suggesting that this adipose tissue compartment directly contributes to the disease process (133).

#### 5.2.1 Leptin and T cell–mediated gut inflammation

Leptin was initially believed to function solely as a satiety factor (134, 135). However, research conducted by Lord and colleagues demonstrated that the adipokine leptin plays a crucial role in promoting T cell proliferation, which results in an increase in type 1 helper T cells (TH1) while simultaneously inhibiting the production of TH2 cytokines (136). Furthermore, studies using experimental autoimmune encephalomyelitis models provide compelling evidence for the regulatory role of leptin in autoimmune diseases (137, 138).

Previous studies have measured systemic leptin concentrations during acute experimental colitis. In the current study, it was found that plasma leptin concentrations were elevated in patients with TNBS-induced colitis and indomethacin-induced ileitis, and these levels correlated with disease severity. Notably, this increase in leptin is transient, with concentrations returning to baseline over time (139). Additionally, an independent study has demonstrated significantly increased leptin expression in mesenteric adipose tissue in patients diagnosed with Crohn’s disease (140, 141).

Previous studies have demonstrated that leptin-deficient ob/ob mice exhibit protection against dextran sulfate sodium (DSS)-induced colitis, whereas administration of leptin renders these mice susceptible to the disease (142). Furthermore, the researchers established a T cell transfer model of colitis, which indicated that the stimulatory effects of leptin play a crucial role in this model, significantly delaying the onset of colitis (143). Additional data show that OB/OB mice are protected in TH1 (trinitrobenzene sulfonic acid-TNBS) or TH_2_ (oxazolone) cell-driven models, which emphasizes that leptin’s T cell stimulating ability is critical for the observed effects (144).

In addition to its effects on epithelial cells, leptin also modulates intestinal inflammation through T cell studies by Sitharaman et al., which showed that inflamed epithelial cells produce leptin and release it into the intestinal lumen. The presence of luminal leptin leads to the activation of the NF-κB pathway in intestinal epithelial cells. Intestinal inflammation and epithelial wall damage will occur after rectal application of leptin (145).

#### 5.2.2 Adiponectin: dual roles in gut immune regulation

Adiponectin, as an important adipokine, is increased in inflammatory bowel disease. The study conducted by Yamamoto et al. examined the expression of adiponectin in the adipose tissue of patients with Crohn’s disease. The findings indicated that adiponectin expression in the adipose tissue of these patients was positively correlated with adiponectin levels in the same tissue. Furthermore, fat content was found to be upregulated in patients with Crohn’s disease compared to those with colitis or healthy controls (146).

Although some experimental models (DSS and TNBS) have shown that adiponectin can induce the production of pro-inflammatory cytokines in the colon (147). For example, globular adiponectin mediated pro-proliferative as well as pro-inflammatory effects through activation of extracellular-signal regulated kinase (ERK), p38 mitogen-activated protein kinase (MAPK), and NF-κB signaling on colonic epithelial cells (148). However, Nishihara verified that adiponectin protects against inflammation, which may be mediated through direct anti-inflammatory effects on colon epithelial cells (149).

A recent study demonstrated that treatment with adiponectin-deficient DSS resulted in more severe colitis, which was associated with an increase in activated B cells in the colon as well as elevated levels of pro-inflammatory cytokines such as IL-1β, IL-4, and IL-6, and enhanced STAT3 signaling. Gene knockout animals exhibited reduced epithelial cell proliferation and increased cellular stress. In *in vitro* experiments, adiponectin was shown to reverse these effects. These findings support the notion that adiponectin remains stable within the intestinal environment (150).

### 5.3 Adipokine signaling pathways linking gut and lung inflammation

Intestinal stability is essential for maintaining host health and preventing IBD. The Wnt/β-Catenin signaling pathway is highly active within the intestinal microenvironment, playing a crucial role in the renewal process of intestinal epithelial cells. In IBD patients, the expression of genes associated with the Wnt/β-catenin signaling pathway is significantly increased, and disruptions in this pathway’s function can induce T cell inflammation, thereby promoting the occurrence of colitis (151).

With increased expression in the Wnt/β-catenin pathway, the Wnt protein levels rise, leading to the activation of the related Wnt/Wingless signaling pathway. Activation of Wnt signaling has been shown to reduce triglyceride (TG) accumulation and increase free fatty acid (FFA) levels by promoting lipolysis and reducing fat production (152).

**FIGURE 3:**
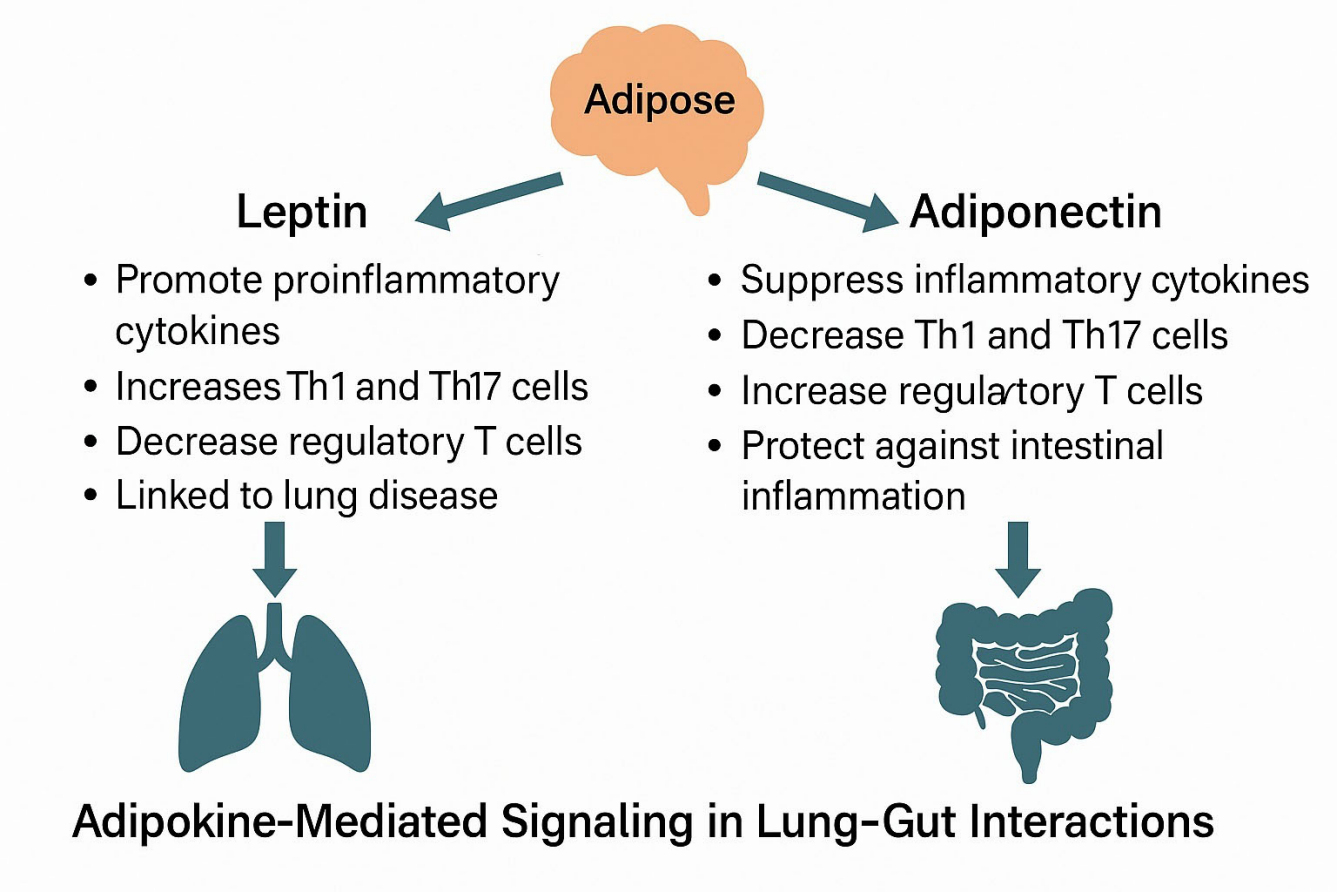
Immunomodulatory roles of leptin and adiponectin in lung–gut crosstalk.

Following lipolysis, lipids stored within adipocytes are released into the bloodstream, where they undergo further processing and stimulate the secretion of leptin. It is clear from the preceding discussion that leptin significantly influences lung-related diseases. Although the Wnt/β-catenin signaling pathway has been implicated in causing pulmonary inflammatory damage (153), limited research exists on whether this pathway affects lung function through adipokines. While adiponectin levels are markedly elevated in IBD patients, adiponectin does not appear to be directly associated with Wnt-related signaling pathways and may independently inhibit inflammation progression. However, limited literature exists regarding adipokine-mediated signaling pathways in the context of lung diseases leading to intestinal diseases. Consequently, further investigation is warranted to clarify the role of adipokines in lung-intestinal interactions. Future studies should explore the mechanisms by which adipokines influence microflora translocation in lung-intestinal interactions (Figure 3).

### 5.4 Limitations and future research directions

While emerging findings suggest that adipokines such as leptin and adiponectin may play immunomodulatory roles in both pulmonary and gastrointestinal contexts, definitive evidence linking these molecules to lung–gut crosstalk remains limited. Most current data are derived from observational human studies, experimental colitis models, or indirect respiratory outcomes in obesity research. Future investigations should aim to directly assess how adipokines influence epithelial barrier function, cytokine signaling, and microbial composition across both organ systems, using integrative multi-omics and targeted genetic models. Furthermore, clinical studies stratifying patients by adipokine profiles and disease comorbidities may offer insight into translational relevance. Moreover, modulation of gut microbiota has been proposed as a potential therapeutic strategy for pulmonary diseases. Zhang et al. summarized probiotic and prebiotic interventions that alleviate airway inflammation, and Li et al. proposed that reshaping gut microbiota may improve response to immunotherapy in lung cancer patients (7, 8). These insights underscore the translational potential of targeting the gut–lung axis. Given the central roles of leptin and adiponectin in lung and gut inflammation, potential therapeutic strategies may include leptin receptor antagonists, adiponectin receptor agonists (e.g., AdipoRon), and agents that modulate the adiponectin/leptin ratio. These approaches have shown promise in preclinical models and may offer novel interventions in lung–gut axis–related diseases, especially in the context of obesity-driven inflammation.

## 6 Conclusion and outlook

The lungs and intestines both originate from the foregut region of the endoderm, which contributes to their functional similarities. Different pathogens elicit comparable immune responses, thereby further supporting the theory of lung-gut interaction. The primary mechanism underlying this interaction focuses on bacterial translocation and the corresponding immune system response.

While mechanical ventilation-induced lung injury and inflammatory bowel disease may complement the potential for pulmonary and intestinal diseases, the underlying causes and consequences of lung and intestinal dysbiosis remain poorly understood. Furthermore, given the widespread distribution of adipose tissue in the human body, adipokines play a significant role in mediating lung-intestinal interactions by influencing the dynamics of lung and intestinal microbiota and immune responses. Additionally, the mechanisms of lung-intestinal interaction associated with other types of lung and intestinal diseases are anticipated to be further elucidated in future research. The role of adipokines in lung-intestinal interactions was further investigated by categorizing patients with varying degrees of obesity and malnutrition.
